# The Incidence of Long-Term Fatigue in Patients Who Achieved Remission From COVID-19 at King Abdulaziz Medical City

**DOI:** 10.7759/cureus.33869

**Published:** 2023-01-17

**Authors:** Waleed M Alotibi, Zaid Alzeer, Ibrahim F Alfarhan, Abdulrahman D Alharbi, Faris A Alhaqbani, Ahmed S Almutairi, Taghreed M Alhazmi

**Affiliations:** 1 Family Medicine, King Saud Bin Abdulaziz University for Health Sciences (KSAU-HS) College of Medicine, Riyadh, SAU; 2 Medicine, King Saud Bin Abdulaziz University for Health Sciences (KSAU-HS) College of Medicine, Riyadh, SAU; 3 Family Medicine and Primary Care, King Abdulaziz Medical City, Riyadh, SAU

**Keywords:** long term, fatigue syndrome, post viral syndrome, covid-19, chronic fatigue

## Abstract

Introduction

Long-term fatigue is a common condition that involves both physical and psychiatric symptoms, and it affects multiple age groups and causes morbidity and disabling symptoms that range from mild to severe symptoms. Many patients are discharged following coronavirus disease 2019 (COVID-19) infection without proper follow-up and evaluation of long-term effects, resulting in the improper treatment of the long-term symptoms, which increases the burden on the patients and healthcare systems. Coronavirus disease 2019 (COVID-19) is a disease caused by the novel SARS-CoV-2. It results in a variety of symptoms, including fever, cough, respiratory distress, the loss of the sense of smell and taste, and long-term effects such as post-severe acute respiratory syndrome (SARS), which is characterized by chronic fatigue, sleep disturbances, myalgia, weakness, and depression. The aim of this study is to assess the incidence of long-term fatigue in patients who achieved remission from COVID-19 at King Abdulaziz Medical City (KAMC), National Guards Health Affairs, Riyadh.

Methods

We conducted a cross-sectional, non-probability convenience sampling study. All participants who were diagnosed with COVID-19 and achieved remission were approached in an outpatient department (OPD) setting and signed an informed consent form and were evaluated by standard questionnaires at clinics after remission from COVID-19 at King Abdulaziz Medical City in Riyadh, Saudi Arabia. A total of 343 subjects who fit the inclusion criteria of any patients who have been diagnosed with COVID-19 and achieved remission were included in the study. This study included patients from the National Guard Hospital, students, and staff members. The primary outcome variable was the incidence of long-term fatigue in patients who achieved remission from COVID-19 as measured by the Chalder fatigue scale (CFQ). The participants were approached in clinics and general OPD by one of the research teams.

Results

Based on the study design, 343 patients were selected from King Abdulaziz Medical City in Riyadh, the incidence of long-term fatigue in patients who achieved remission from COVID-19 was 55.7%, and the rest were normal (44.3%). The incidence of long-term fatigue was statistically significantly higher in females and those who had been diagnosed with COVID-19 and achieved remission for more than two months. The age of the participants ranged from 18 to more than 45, with a predominance of females (60.6%). Regarding body mass index (BMI), 39.9% were overweight, and 29.2% were obese. Additionally, the incidence of patients with associated chronic disease was 27.4%; among these chronic diseases, hypertension was the most common one (18.1%), followed by diabetes (17%) and thyroid diseases (14.9%).

Conclusion

To the best of our knowledge, this is one of the few studies that were carried out in Saudi Arabia that assess long-term fatigue post COVID-19 infection. In our study, we discovered that long-term fatigue was highly prevalent (55.7%). We found that among those participants, more than half of those who reported chronic fatigue had a COVID-19 diagnosis for longer than two months. Furthermore, females made up the majority of those who had long-term fatigue. We urge that additional longitudinal and standardized studies be carried out in order to thoroughly determine the severity of long-term fatigue in patients who obtained remission from COVID-19.

## Introduction

Long-term fatigue is a common condition that has symptoms of both physical and mental health. It affects people of all ages and can cause morbidity and incapacitating symptoms that can range in severity from mild to severe. It is characterized by unexplained and persistent post-exertional fatigue accompanied by a variety of symptoms related to cognitive, immunological, endocrinological, and autonomous dysfunction [1]. The exact mechanisms of long-term fatigue are not quite understood, with no known causative factor that has been established. However, there is evidence that suggests that infections and immunological dysfunction contribute to the development and maintenance of symptoms and possibly the involvement of genetic and psychosocial factors [1].

Post-infection fatigue has been an emerging long-term consequence of past epidemics. For example, in the 2003 severe acute respiratory syndrome coronavirus (SARS-CoV), the studies have found that the majority of the participants reported fatigue; 64% of them reported fatigue at three months, 54% at six months, and 60% at 12 months [2]. According to another study that has been conducted in Norway in 2015, it was reported that the incidence rate of chronic fatigue syndrome (CFS) following the H1N1 pandemic was 2.08 per 100,000. The incidence was greater in the younger population suggesting that younger age is a considerable risk factor for developing myalgic encephalomyelitis (ME)/chronic fatigue syndrome [3]. It also has been found that post-infection fatigue can occur in a non-epidemic setting, for example, infectious mononucleosis and bacterial infection. Continuous fatigue lasting for six months or longer without a known cause is termed myalgic encephalomyelitis (ME)/chronic fatigue syndrome (CFS). It has been suggested that ME/CFS may be caused by several viral and bacterial infections [4]. Even though it has been established that there is an association between depression and CFS, it is still not known which of the two precedes the other [5,6]. Additionally, while it is believed that certain infections can exacerbate CFS, the exact pathophysiology still remains unclear.

Following a coronavirus disease 2019 (COVID-19) infection, many patients are discharged without receiving the required follow-up care or having their long-term consequences evaluated. This leads to incorrect management of long-term symptoms, which puts an additional burden on patients and healthcare systems. COVID-19 is a disease caused by SARS-CoV-2. It results in a variety of symptoms, including fever, cough, respiratory distress, the loss of the sense of smell and taste, and long-term effects such as post-severe acute respiratory syndrome (SARS), which is characterized by chronic fatigue, sleep disturbances, myalgia, weakness, and depression [7]. There have been several studies that attempted to establish a correlation between long-term fatigue and COVID-19. A study that has been conducted in 2021 in Tehran on 120 COVID-19-positive patients, by using the Fukuda criteria, stated that the prevalence rate of long-term fatigue was 17.5%, while 82.5% of respondents did not report any symptoms [8]. Another study that was conducted in London in 2020 on 384 participants stated that the most common persistent post-discharge symptom was fatigue, accounting for 69% of the patients [9]. According to a study that was conducted in China in 2021 with a population of 1,733, the prevalence rate of fatigue was 63% [10]. The aim of this study was to assess the incidence of long-term fatigue in patients who achieved remission from COVID-19 at King Abdulaziz Medical City (KAMC), National Guards Health Affairs, Riyadh.

## Materials and methods

We conducted a cross-sectional, non-probability convenience sampling study. Participants who were diagnosed with COVID-19 and achieved remission were approached in an outpatient department (OPD) setting and signed an informed consent form and were evaluated by standard questionnaires (Chalder fatigue scale {CFQ}) at clinics after remission from COVID-19 at King Abdulaziz Medical City in Riyadh, Saudi Arabia. A total of 343 subjects who fit the inclusion criteria were included in the study. The participants were approached in clinics and general OPD by one of the research teams. Any adult who has been diagnosed with COVID-19 and achieved remission was included in the study. Additionally, we excluded any patient who has a history of previous life-threatening infection (sepsis, endocarditis, meningitis, or other central nervous system infections) prior to the confirmation of the diagnosis of COVID-19, patients who have received psychiatric medication in the last year, and patients who have been diagnosed of chronic fatigue syndrome prior to infection. Data were collected through a validated questionnaire (CFQ). Patients were informed that their participation is voluntary and their responses will be kept confidential.

A total of six questions regarding the demographics were asked followed by two questions regarding general and mental health. Additionally, there were six questions regarding COVID-19 infection. The main outcome variable was to measure the incidence of long term-fatigue after remission from COVID-19 using the Chalder fatigue scale (CFQ); the scale is composed of 11 items: Do you have problems with tiredness? Do you need to rest more? Do you feel sleepy or drowsy? Do you have problems starting things? Do you lack energy? Do you have less strength in your muscles? Do you feel weak? Do you have difficulties concentrating? Do you make slips of the tongue when speaking? Do you find it more difficult to find the right word? How is your memory?

The Chalder fatigue scale’s scoring method allows for “bimodal” scoring, with columns denoting 0, 0, 1, and 1 with a range from 0 to 11. Alternative scoring options include “Likert”-type scores of 0, 1, 2, and 3, which have a range of 0 to 33. Answers were measured using the Likert scale (0-3). A total of 33 points can be derived from this, along with scores for the physical (measured by items 1-7) and psychological (measured by items 8-11) fatigue subscales [11]. Additionally, the CFQ allowed for the distinction between “cases” and “non-cases” (scores of 4 or higher and less than 4, respectively), where scores 0 and 1 (“Better than usual” and “No worse than usual”) were given a 0 and scores 2 and 3 (“Worse than usual” and “Much worse than usual”) were given a 1 (bimodal scoring). Each of the 11 binary scores was added together, and individuals scoring 4 or higher were within the fatigue criteria. Additionally, individuals with a score of 9 or higher were diagnosed with chronic fatigue syndrome [11]. Permission was obtained from the original publisher to use the Chalder fatigue scale (CFQ).

Descriptive data in this study were presented using numbers and percentages. A p-value cutoff point of 0.05 at 95% confidence interval (CI) was used to determine statistical significance. The analyses measured the relationship between the socio-demographic characteristics and the long-term fatigue by using chi-square test. Multivariate regression analysis had been performed as well to determine the independent predictor associated with the long-term fatigue with corresponding odds ratio, as well as 95% confidence interval. All statistical analyses were performed using the Statistical Package for Social Sciences (SPSS) version 26 (IBM SPSS Statistics, Armonk, NY).

## Results

Based on the study design, 343 patients were selected from King Abdulaziz Medical City in Riyadh; the average age of the participants ranges from 18 to 65, with a predominance of females (60.6%). Regarding body mass index (BMI), 39.9% were overweight, and 29.2% were obese; nearly two-thirds were married (63.3%). Patients who were employed constituted 48.1%, and the incidence of patients with associated chronic disease was 27.4% (Table 1).

**Table 1:** Socio-demographic characteristics of the patients (N=343) N, number; BMI, body mass index

Study variables	N (%)
Age group	
18-25 years	96 (28.0%)
26-35 years	64 (18.7%)
36-45 years	88 (25.7%)
>45 years	95 (27.7%)
Gender	
Male	135 (39.4%)
Female	208 (60.6%)
BMI level	
Normal (18.5-24.9 kg/m^2^)	106 (30.9%)
Overweight (25-29.9 kg/m^2^)	137 (39.9%)
Obese (≥30 kg/m^2^)	100 (29.2%)
Marital status	
Unmarried	126 (36.7%)
Married	217 (63.3%)
Employment status	
Student	74 (21.6%)
Employed	165 (48.1%)
Unemployed	104 (30.3%)
Associated chronic diseases	
Yes	94 (27.4%)
No	249 (72.6%)

**Figure 1:**
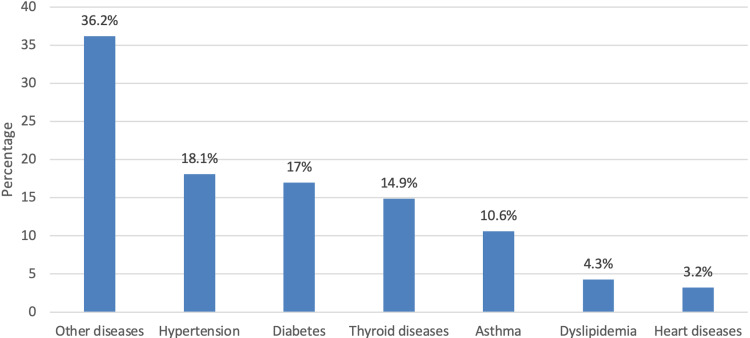
Specific chronic disease of the patients

Figure 1 depicts the specific type of chronic disease as reported by the patients. It can be observed that the most commonly known chronic disease was hypertension (18.1%), followed by diabetes (17%) and thyroid diseases (14.9%) (Figure 1).

More than half (51.9%) had been diagnosed with COVID-19 and achieved remission one to two months ago, where patients mostly exhibited mild conditions (95.9%). Only 2.3% were admitted, while 63.8% expressed that they had been vaccinated prior to being infected by the virus. Of those who had been vaccinated (n=219), 63% received the second dose, and the most common type of received vaccine was Pfizer (85.4%) (Table 2).

**Table 2:** Clinical characteristics of the patients who had been diagnosed with COVID-19 (N=343) N, number; ICU, intensive care unit; COVID-19, coronavirus disease 2019

Variables	N (%)
When were you diagnosed with COVID-19?	
1-2 months	178 (51.9%)
3-4 months	15 (04.4%)
5-6 months	18 (05.2%)
7- 8 months	26 (07.6%)
9-10 months	22 (06.4%)
11-12 months	84 (24.5%)
How severe was your condition?	
Mild (home)	329 (95.9%)
Moderate (hospital)	09 (02.6%)
Severe (ICU)	05 (01.5%)
Were you admitted to the hospital?	
Yes	08 (02.3%)
No	335 (97.7%)
Have you been vaccinated at the time you caught the infection?	
Yes	219 (63.8%)
No	127 (36.2%)
Number of doses received by immunized patients (n=219)	
1st dose	34 (15.5%)
2nd dose	138 (63.0%)
3rd dose	47 (21.5%)
Type of vaccine received (n=219)	
Pfizer	187 (85.4%)
AstraZeneca	18 (08.2%)
Moderna	01 (0.30%)
Mixed vaccine	13 (05.9%)

When measuring the relationship between the level of long-term fatigue and the socio-demographic characteristics of the patients, it was found that the incidence of long-term fatigue was statistically significantly higher in females when compared to males (p=0.013). It was also found to be higher in employed patients when compared to unemployed (p=0.018) and those who had been diagnosed with COVID-19 and achieved remission for more than two months (p=0.028) (Table 3).

**Table 3:** Relationship between the level of long-term fatigue and the socio-demographic characteristics of the patients (N=343) N, number; BMI, body mass index; COVID-19, coronavirus disease 2019

Factors	Level of fatigue		P-value
	Fatigue, N (%) (n=191)	Not fatigue, N (%) (n=152)	
Age group			
≤35 years	92 (48.2%)	68 (44.7%)	0.527
>35 years	99 (51.8%)	84 (55.3%)	
Gender			
Male	64 (33.5%)	71 (46.7%)	0.013
Female	127 (66.5%)	81 (53.3%)	
BMI level			
Normal	61 (31.9%)	45 (29.6%)	0.675
Overweight	78 (40.8%)	59 (38.8%)	
Obese	52 (27.2%)	48 (31.6%)	
Marital status			
Unmarried	71 (37.2%)	55 (36.2%)	0.850
Married	120 (62.8%)	97 (63.8%)	
Employment status			
Student	44 (23.0%)	30 (19.7%)	0.018
Employed	101 (52.9%)	64 (42.1%)	
Unemployed	46 (24.1%)	58 (38.2%)	
Associated chronic diseases			
Yes	54 (28.3%)	40 (26.3%)	0.687
No	137 (71.7%)	112 (73.7%)	
Diagnosed with COVID-19 since			
1-2 months	89 (46.6%)	89 (58.6%)	0.028
>2 months	102 (53.4%)	63 (41.4%)	
Had been vaccinated at the time caught by the infection			
Yes	121 (63.4%)	98 (64.5%)	0.830
No	70 (36.6%)	54 (35.5%)	

In a multivariate regression model (Table 4), it was observed that compared to males, the odds of having long-term fatigue could likely increase in females by at least 2.1 times higher (adjusted odds ratio {AOR}: 2.126; 95% CI: 1.309-3.454; p=0.002). Also, patients who had been diagnosed with COVID-19 more than two months ago were predicted to increase the risk of long-term fatigue by at least 1.7% higher as compared to those who had been diagnosed with COVID-19 earlier (AOR: 1.669; 95% CI: 1.070-2.601; p=0.024). However, compared to students, patients who were employed were predicted to have an increased risk of long-term fatigue by at least 64% (AOR: 0.358; 95% CI: 0.184-0.698; p=0.003), while for those unemployed patients, the odds might be decreased by at least 55% (AOR: 0.448; 95% CI: 0.267-0.749; p=0.002) (Table 4).

**Table 4:** Multivariate regression analysis to determine the independent factor associated with long-term fatigue (N=343) AOR, adjusted odds ratio; CI, confidence interval; COVID-19, coronavirus disease 2019

Factors	AOR	95% CI	P-value
Gender			
Male	Reference		
Female	2.126	1.309-3.454	0.002
Employment status			
Student	Reference		
Employed	0.358	0.184-0.698	0.003
Unemployed	0.448	0.267-0.749	0.002
Diagnosed with COVID-19 since			
1-2 months	Reference		
>2 months	1.669	1.070-2.601	0.024

## Discussion

This study aimed to assess the incidence of long-term fatigue in patients who achieved remission from COVID-19 at King Abdulaziz Medical City, National Guards Health Affairs, Riyadh, which was (55.7%) among the subjects who achieved remission using the Chalder fatigue scale. We have found similar results in a meta-analysis study that was conducted in 2021 in the USA, which showed that the most common long-term effect of COVID-19 was fatigue, accounting for 58% of the long-term manifestations [12]. On the other hand, we have found another meta-analysis study that was conducted in China demonstrating lower prevalence rates of 36% [13]. There has been a well-established association between chronic fatigue syndrome and several viral infections, such as Epstein-Barr virus (EBV), cytomegalovirus, enterovirus, and herpesvirus, and according to the results of our study and many other studies, we can assume that COVID-19 carries an increased risk of developing long-term fatigue [14]. Additionally, in regard to the association of long-term fatigue with a history of COVID-19, we have found that patients who achieved remission for more than two months were at higher risk of developing long-term fatigue, accounting for an increase of 1.7% higher than those who achieved remission later (1-2 months) (Table 4). Contrary to another research, 53% of hospitalized patients reported fatigue on an average of 36 days after release (60 days after symptom onset) (n=143) [15], while 60%-70% of patients reported fatigue on an average of 48 days after hospital discharge [16]. On the other hand, there was no significant association between those who were vaccinated and those who were not regarding the risk of developing long-term fatigue (p=0.830) (Table 3). Furthermore, there was no association between chronic diseases and the likely hood of developing long-term manifestations (p=0.687).

Identification of patients’ characteristics and other factors associated with long-term fatigue

In our study, we found that in regard to patients’ characteristics and association with long-term fatigue, there was no significant difference in age groups in relation to the development of long-term fatigue (p=0.527) (Table 3). Moreover, we found that the incidence of long-term fatigue was statistically significant and higher in females (p=0.013), with a ratio of 2:1 for female patients. This result was similar to another study that stated that females are at a higher risk of developing long-term fatigue when compared to males [17]. This could be due to the fact that the majority of participants are females in both studies, which may be due to the fact that most of the patients that we encountered at the OPD clinics were females. Another statistically significant association we found was that patients who were employed were predicted to have an increased risk of long-term fatigue by at least 64% (AOR: 0.358; 95% CI: 0.184-0.698; p=0.003), while for those who are unemployed, the odds might be decreased by at least 55%.

In our study, we have found that the majority of participants received Pfizer vaccine, accounting for 85% when compared to other vaccines such as AstraZeneca (8.2%) and Moderna (0.3%). This could be due to the fact that Pfizer was more available and started to operate earlier than AstraZeneca and Moderna in the Kingdom of Saudi Arabia (KSA). Another factor that could be related to the difference is media. Pfizer was more encouraged to be taken by influencers on social media without any scientific evidence that proves that Pfizer is much better in regard to providing immunity from COVID-19. Thus, more campaigns for raising awareness and educating people not to follow whatever is said on social media are highly advised. Another factor that has been reported frequently by people who took the later vaccines is side effects such as injection site redness, fever, headache, and muscular pain. People who took AstraZeneca were more susceptible to develop side effects when compared to people who received Pfizer vaccine [18].

If only treating medical physicians are consulted for follow-up, the management of tiredness states will not be properly handled. To treat CFS and perhaps address post-infection fatigue, a variety of therapies are required, including graded exercise and cognitive behavioral therapy [19,20]. Additionally, employers and occupational health agencies will need to continue to provide input for a successful return to work [21].

Limitations

There are various limitations to our single-center study that was conducted on staff members, patients, and students in King Abdulaziz Medical City (KAMC), National Guards Health Affairs, Riyadh; that should be discussed. We only evaluated individuals at a one-time point in our cross-sectional research.

## Conclusions

In our study, we found that the incidence of long-term fatigue was high (55.7%). Among those participants, we found that more than half of the participants who experienced long-term fatigue were diagnosed with COVID-19 for more than two months. Additionally, the majority of the participants who experienced long-term fatigue were females. As this is the first local study to assess long-term fatigue in patients who achieved remission from COVID-19, we recommend that further longitudinal and standardized studies be conducted to fully assess the severity of long-term fatigue. Worldwide, a sizable proportion of patients are making a full recovery from SARS-CoV-2 infection. Long-term post-infection fatigue can negatively affect the quality of life and have a big impact on individuals, employers, and healthcare systems. These significant findings draw attention to a developing problem of an increased burden of COVID-19. These results should be incorporated into recovery patient treatment plans to facilitate prompt action. Furthermore, we recommend a thorough follow-up for those who achieved remission from COVID-19, in regard to the severity of the fatigue symptoms and possible work impairment.

## Appendices

The six questions regarding COVID-19 infection are shown in Tables 5-6.

**Table 5:** Demographic information

How old are you?	______		
What is your gender?	Male ( )	Female ( )	
What is your height?	______ cm		
What is your weight?	______ kg		
What is your marital status?	Married ( )		
	Separated ( )		
	Widowed ( )		
	Divorced ( )		
	Never married ( )		
What is your employment status?	On disability ( )		
	Student ( )		
	Homemaker ( )		
	Retired ( )		
	Unemployed ( )		
	Working part time ( )		
Have you been diagnosed with any condition?	Yes ( )	No ( )	If yes, what? ____________
Did you receive any psychiatric medications in the past year?	Yes ( )	No ( )	

**Table 6:** History of COVID-19 COVID-19, coronavirus disease 2019; ICU, intensive care unit

History of COVID-19						
1. When were you diagnosed with COVID-19?	1 month	2-3 months	4-6 months	7-8 months	9-10 months	11-12 months
2. How severe was your condition?	Mild (home)	Moderate (hospital)	Severe (ICU)			
3. Were you admitted in the hospital?	Yes (how long? ___)	No				
4. Have you been vaccinated at the time you caught the infection? (if no, skip questions 5-6)	Yes	No				
5. If question 4 is yes, were you fully immunized or not?	First dose	Second dose	Third dose			
6. If question 4 is yes, which vaccine did you take?	Pfizer	AstraZeneca	Moderna	Mixed vaccines		

Table 7 shows the 11 items in the Chalder fatigue scale.

**Table 7:** Chalder fatigue scale This scale can be scored “bimodally” with columns representing 0, 0, 1, and 1 and a range from 0 to 11 with a total of 4 or more qualifying for “caseness.” Alternatively, it can be scored in “Likert” style 0, 1, 2, and 3 with a range from 0 to 33. The mean “bimodal” score for CFS sufferers was 9.14 (SD: 2.73) and for a community sample 3.27 (SD: 3.21). The mean “Likert” score was 24.4 (SD: 5.8) and 14.2 (SD: 4.6) (total: 0-33) Source: This study involved 361 CFS sufferers and 1,615 individuals from the community. The average age was in the 30s. Fatigue levels were similar for males and females. A score of 29 discriminated between CFS sufferers and the community sample in 96% of cases, and a score in the 30s discriminated in 100% of cases. The CFS sufferers also scored a mean of 26.99 on the Work and Social Adjustment Scale (WSAS) with an SD of 8.6 (i.e., about 70% scoring between 18.4 and 35.6) [22] CFS: chronic fatigue syndrome

Chalder fatigue scale				
	Less than usual	No more than usual	More than usual	Much more than usual
Do you have problems with tiredness?				
Do you need to rest more?				
Do you feel sleepy or drowsy?				
Do you have problems starting things?				
Do you lack energy?				
Do you have less strength in your muscles?				
Do you feel weak?				
Do you have difficulties concentrating?				
Do you make slips of the tongue when speaking?				
Do you find it more difficult to find the right word?				
	Better than usual	No worse than usual	Worse than usual	Much worse than usual
How is your memory?				
